# Genetic Hearing Loss Associated With Autoinflammation

**DOI:** 10.3389/fneur.2020.00141

**Published:** 2020-03-05

**Authors:** Hiroshi Nakanishi, Pragya Prakash, Taku Ito, H. Jeffrey Kim, Carmen C. Brewer, Danielle Harrow, Isabelle Roux, Seiji Hosokawa, Andrew J. Griffith

**Affiliations:** ^1^Department of Otorhinolaryngology/Head & Neck Surgery, Hamamatsu University School of Medicine, Hamamatsu, Japan; ^2^Otolaryngology Branch, National Institute on Deafness and Other Communication Disorders, National Institutes of Health, Bethesda, MD, United States; ^3^Department of Otorhinolaryngology, Tokyo Medical and Dental University, Tokyo, Japan; ^4^Office of the Clinical Director, National Institute on Deafness and Other Communication Disorders, National Institutes of Health, Bethesda, MD, United States

**Keywords:** cryopyrin-associated periodic syndromes, hearing loss, interleukin-1β, macrophage, *NLRP3*, Pendred syndrome, *SLC26A4*

## Abstract

Sensorineural hearing loss can result from dysfunction of the inner ear, auditory nerve, or auditory pathways in the central nervous system. Sensorineural hearing loss can be associated with age, exposure to ototoxic drugs or noise, or mutations in nuclear or mitochondrial genes. However, it is idiopathic in some patients. Although these disorders are mainly caused by dysfunction of the inner ear, little of the pathophysiology in sensorineural hearing loss is known due to inaccessibility of the living human inner ear for biopsy and pathological analysis. The inner ear has previously been thought of as an immune-privileged organ. We recently showed that a missense mutation of the *NLRP3* gene is associated with autosomal-dominant sensorineural hearing loss with cochlear autoinflammation in two unrelated families. *NLRP3* encodes the NLRP3 protein, a key component of the NLRP3 inflammasome that is expressed in immune cells, including monocytes and macrophages. Gain-of-function mutations of *NLRP3* cause abnormal activation of the NLRP3 inflammasome leading to IL-1β secretion in a spectrum of autosomal dominant systemic autoinflammatory phenotypes termed cryopyrin-associated periodic syndromes. The affected subjects of our two families demonstrated atypical phenotypes compared with those reported for subjects with cryopyrin-associated periodic syndromes. These observations led us to test the hypothesis that macrophage/monocyte-like cells in the cochlea can mediate local autoinflammation via activation of the NLRP3 inflammasome. The inflammasome can indeed be activated in macrophage/monocyte-like cells of the mouse cochlea, with secretion of IL-1β. The macrophage/monocyte-like cells in the cochlea were also found to be associated with hearing loss in a *Slc26a4*-insufficient mouse model of human deafness. This review addresses our understanding of genetic hearing loss mediated by autoinflammation and macrophage/monocyte-like cells in the cochlea.

## Introduction

Sensorineural hearing loss can result from dysfunction of the inner ear, auditory nerve or higher auditory pathways in the central nervous system. Sensorineural hearing loss includes a wide variety of disorders, such as idiopathic sudden sensorineural hearing loss, age-related hearing loss, hearing loss associated with exposure to ototoxic drugs or noise, and almost all heritable forms of non-syndromic hearing loss. Although these disorders are usually caused by dysfunction of the inner ear, the pathophysiology in sensorineural hearing loss is usually unknown due to inaccessibility of the living human inner ear for biopsy and pathological analysis.

In this review, we focus on the hearing loss caused by dysregulation of the NLRP3 inflammasome. The NLRP3 inflammasome is a critical component of a widely studied, canonical inflammatory signaling pathway in the innate immune system. We review syndromic and non-syndromic hearing loss phenotypes caused by an *NLRP3* mutation. We also review the role of cochlear macrophages in a mouse model of genetic hearing loss phenotypes, non-syndromic recessive deafness DFNB4 and Pendred syndrome, caused by variants of the *SLC26A4* gene.

## NLRP3 Inflammasome and ITS Activation Pathway

**Figure 1:**
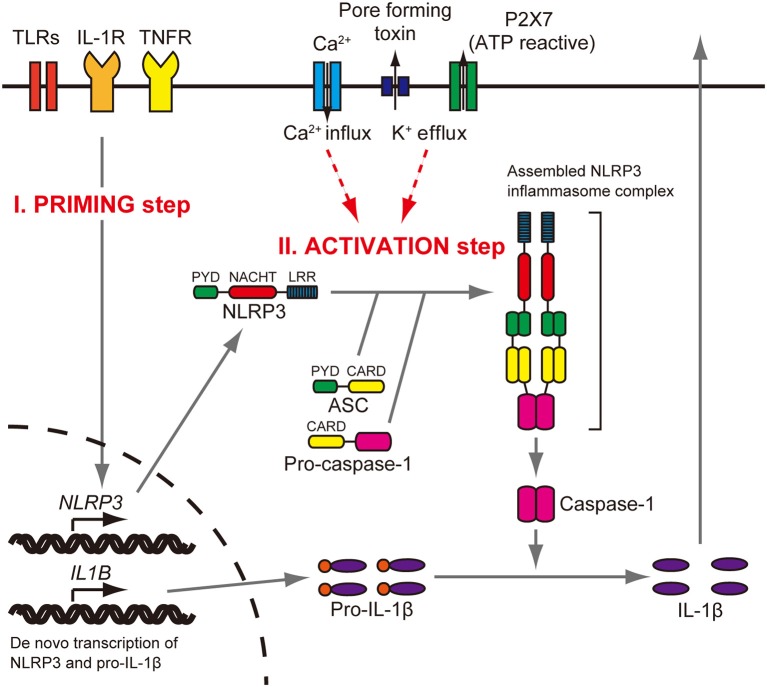
NLRP3 inflammasome activation. Stimulation of Toll-like receptors (TLRs), IL-1 receptor (IL-1R), or tumor necrosis factor receptor (TNFR) increases NLRP3 and pro–IL-1β protein expression (priming step). The activation step involves efflux of K^+^, which can be initiated by pore-forming toxin or ATP-triggered channel P2X7, or Ca^2+^ influx. When the NLRP3 inflammasome is activated, the PYD domain mediates recruitment of ASC and procaspase-1 to form a complex that cleaves inactive procaspase-1 to generate active caspase-1. Caspase-1 can process pro–IL-1β to mature IL-1β, which is secreted. Reproduced from Figure S1, Nakanishi et al. (6).

The *NLRP3* gene (NLR family, pyrin domain containing three, MIM 606416) encodes the NLRP3 protein (also called cryopyrin), a key and eponymous component of the NLRP3 inflammasome (1). The NLRP3 inflammasome is an innate immune sensor expressed in immune cells, such as monocytes, macrophages, and dendritic cells (2–4). The NLRP3 protein consists of an N-terminal pyrin domain (PYD), a central nucleotide-binding oligomerization (NACHT) domain, and a leucine-rich repeat (LRR) domain at the C terminus (5). When the NLRP3 inflammasome is activated, the PYD domain mediates recruitment of ASC (apoptosis-associated speck-like protein containing CARD) and procaspase-1 to form an NLRP3 inflammasome complex that cleaves inactive procaspase-1 to form active caspase-1 (Figure 1). Caspase-1 can process pro–IL-1β to mature IL-1β, a secreted proinflammatory cytokine (1, 7, 8). Activation of the NLRP3 inflammasome requires at least two signals (9). The initial priming signal includes Toll-like receptor ligands, such as bacterial lipopolysaccharide (LPS), that result in increased NLRP3 and pro–IL-1β mRNA and protein expression (3). The second signal can be one of a variety of activators that include adenosine triphosphate (ATP), extracellular calcium, crystalline molecules, or pore-forming toxins (10, 11). Other second signals include heme, or pathogen associated RNA (12, 13), with further details provided in a recent review (14).

## Mutations of *Nlrp3* Cause Cryopyrin-Associated Periodic Syndromes (Caps)

Gain-of-function mutations of *NLRP3* cause a spectrum of autosomal-dominant systemic autoinflammatory diseases called cryopyrin-associated periodic syndromes (CAPS). The CAPS spectrum includes three classical clinical subtypes: neonatal-onset multisystem inflammatory disease (NOMID, MIM 607115), Muckle-Wells syndrome (MWS, MIM 191900), and familial cold autoinflammatory syndrome (FCAS, MIM 120100). These phenotypes share signs and symptoms including recurrent fever, rash, headache, conjunctivitis, and arthralgia or arthritis. All of the CAPS subtypes include serologic evidence of systemic inflammation.

*NLRP3* mutations lead to CAPS via constitutive activation of the NLRP3 inflammasome and increased IL-1β production (5, 15–18). Monocytes from patients with *NLRP3* mutations only require the initial priming signal, not a second activating signal, to induce IL-1β secretion (15). IL-1β activates cells by binding and signaling through IL-1 receptor type I and the IL-1 receptor accessory protein. Anakinra, a non-glycosylated recombinant version of the endogenous human IL-1 receptor antagonist, significantly improves the clinical signs and symptoms and inflammatory markers of NOMID, MWS, and FCAS (19–21).

## Auditory Phenotypes Associated With Caps

Hearing loss is one of the most common manifestations of CAPS (20). Ahmadi et al. reported the prevalence of hearing loss to be 76, 86, 33, and 25% in NOMID, NOMID/MWS, MWS, and FCAS subjects, respectively, among a cohort of 57 patients diagnosed with CAPS (22). Hearing loss was characteristically worse in the higher frequencies and appeared to progress with age. Sensorineural hearing loss was the most common type of hearing loss in all groups: 61% of NOMID, 71% of NOMID/MWS, 33% of MWS, and 25% of FCAS patients.

On MRI- FLAIR examination, cochlear enhancement was detected in 90% of NOMID, 55% of NOMID/MWS, 33% of MWS and 17% of FCAS patients (22). Cochlear enhancement is thought to represent diffusion of contrast material into cochlear tissues from blood vessels comprising the blood-labyrinth barrier that are rendered permeable by inflammation (20, 22).

There are several reports of hearing loss caused by NLRP3-mediated inflammation that was successfully treated with anakinra, an IL-1 receptor antagonist (19–21). After 60 months of anakinra therapy for 26 patients with NOMID, the hearing improved in 30% of ears (23). Hearing loss was stabilized with anakinra therapy in the majority of the patients, but 13 ears developed further hearing loss over 60 months. Cochlear enhancement on MRI-FLAIR was initially observed in 22 of 25 patients. Cochlear enhancement was reduced over 60 months but it was persistent in 14 of 25 patients at 36 months and 10 of 19 patients at 60 months. The incomplete hearing improvement in response to anakinra therapy likely reflects irreversible cochlear damage from prior chronic inflammation, so prompt initiation of therapy at or soon after the onset of SNHL might be essential for preventing or reversing cochlear damage from uncontrolled NLPR3-mediated autoinflammation.

## An *NLRP3* Mutation Causes Non-Syndromic and Syndromic Hearing Loss

We recently reported a gain-of-function mutation, c.2753G > A (p.Arg918Gln) in *NLRP3*, that causes autosomal dominant non-syndromic hearing loss (DFNA34) in a North American Caucasian family (LMG113) (6). There were no symptoms or physical signs of systemic inflammation. Their hearing loss was symmetric, bilateral, and progressive. The age of onset varied from the second to fourth decade of life. Post-contrast MRI-FLAIR examination of the temporal bones of two affected members of LMG113 revealed pathologic enhancement of the cochlea that was similar but less severe than that observed in NOMID or MWS patients with sensorineural hearing loss. These results indicate that the subjects had sensorineural hearing loss associated with radiologic evidence of cochlear inflammation.

The c.2753G > A (p.Arg918Gln) mutation of *NLRP3* also causes sensorineural hearing loss with mixed signs and symptoms of systemic autoinflammation in affected members of an unrelated North American family, LMG446, of mixed Caucasian and Hispanic ancestry (6). The 35 year-old father had a history of progressive bilateral sensorineural hearing loss and symptoms of systemic autoinflammation. His three offspring all carried the p.Arg918Gln mutation and had symptoms and signs of systemic autoinflammation. Thus, the affected members of LMG446 had systemic autoinflammatory phenotypes, but none of them met diagnostic criteria for NOMID, MWS, or FCAS. One sibling (13 yo) had bilateral hearing loss at high frequencies, another (10 yo) had right-sided hearing loss at high frequencies, whereas the youngest (6 yo) had hearing thresholds within normal limits, that may reflect his young age and presymptomatic status. MRI-FLAIR pre-contrast evaluations demonstrated abnormally increased signal in all the affected subjects. This finding was considered to reflect probable evidence of prior inflammation. Family LMG446 thus co-segregates the p.Arg918Gln mutation of *NLRP3* with a novel atypical form of CAPS.

## IL-1β Blockade Reverses Hearing Loss in Family LMG446

Three affected members of family LMG446 were treated with subcutaneous anakinra. After 5 months of therapy, the pure-tone audiometric thresholds of two members were completely within normal limits (6). The thresholds of the third family member improved to within the range of age- and sex-adjusted normative thresholds for the left ear. The improvements in hearing correlated with a decrease in MRI-FLAIR signals. These results indicate that hearing loss is associated with cochlear inflammation caused by NLRP3 inflammasome activation.

## Inner Ear Macrophages Express *NLRP3*

All of these observations strongly implicate cochlear inflammation in the pathogenesis of hearing loss. This raises the question of whether the hearing loss is secondary to systemic autoinflammation with cochlear infiltration of circulating immune cells, or primary autoinflammation with pathologic activation of the NLRP3 inflammasome within the resident immune cells of the cochlea.

**Figure 2:**
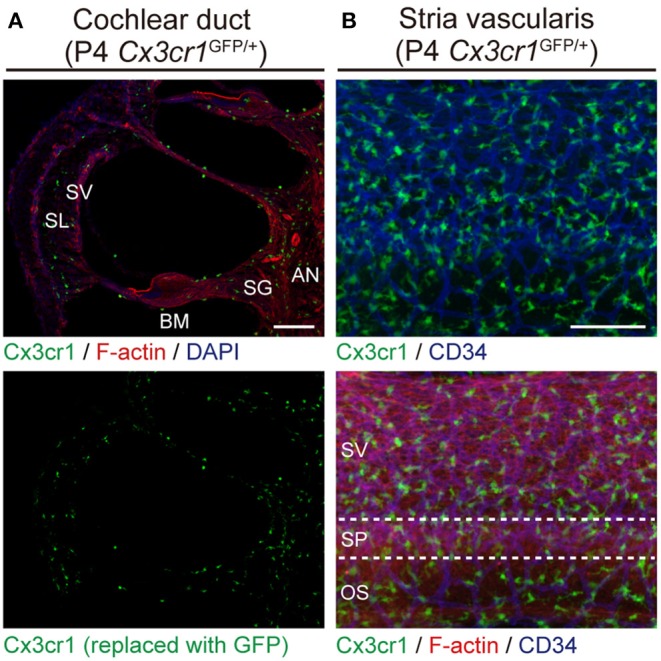
Resident macrophage-like cells in mouse cochlea. **(A)** P4 *Cx3cr1*^GFP/+^ cochleae have GFP^+^ cells scattered throughout the cochlea, including the auditory nerve (AN), spiral ganglion (SG), basilar membrane (BM), stria vascularis (SV), and spiral ligament (SL). Phalloidin (red) and DAPI (blue) label F-actin and nuclei, respectively. **(B)** Lateral wall of a P4 *Cx3cr1*^GFP/+^ cochlea with GFP^+^ cells mainly localized around blood vessels (blue, labeled with anti-CD34 antibody). SV, stria vascularis; SP, spiral prominence; OS, outer sulcus; scale bars, 100 μm. Reproduced from Figure 7, Nakanishi et al. (6). GFP^+^ cells are similarly distributed in all parts of the cochlea at P30 (6).

*Cx3cr1*^GFP^ mice can be used to identify monocytes, as well as subsets of NK cells, dendritic cells, and resident macrophages (24). Adult *Cx3cr1*^GFP/GFP^ mouse cochleae have GFP^+^ cells which also express a lymphocyte marker CD45 and macrophage markers CD68 and Iba1 (25). We also found GFP^+^ cells scattered throughout the auditory nerve, spiral ganglion, basilar membrane, stria vascularis, and spiral ligament (Figure 2) (6). The GFP^+^ cells were primarily localized adjacent to or near blood vessels in the lateral wall and basilar membrane (Figure 2). These GFP^+^ cells also express the macrophage marker F4/80. These studies demonstrate that these GFP^+^ cells are tissue-resident macrophage-like cells that exist in the adult mouse cochlea.

We have shown that *Nlrp3* is expressed in cochlea-resident cells expressing *Cx3cr1* (6). *Nlrp3* mRNA was detected in GFP^+^ cells but not in GFP^−^ cells, indicating that normal mouse cochlear macrophage-like cells express *Nlrp3*. We did not try to detect or analyze NLRP3 protein since we were unaware of any commercial or custom antibodies that were specific.

## NLRP3 Inflammasome Can Be Activated in Inner Ear Macrophages

**Figure 3:**
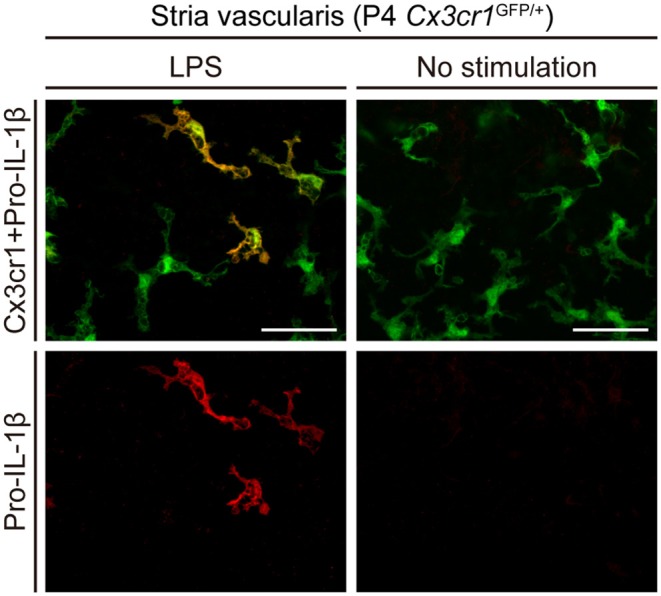
Cultured lateral wall of a P4 *Cx3cr1*^GFP/+^ cochlea stained with anti-IL-1β antibody (red). Pro-IL-1β immunoreactivity is present in a subset of GFP^+^ cells from cultured cochleae stimulated with LPS, whereas no immunoreactivity is detected without stimulation. Scale bars, 50 μm. Reproduced from Figure 10, Nakanishi et al. (6).

To determine if the NLRP3 inflammasome can be activated in wild-type mouse cochleae (postnatal day 3–4, C57BL/6J mouse strain), we measured levels of IL-1β in supernatants of cultured wild-type mouse cochlear tissues. Higher levels of IL-1β were secreted from cultured cochleae stimulated with LPS and ATP, in comparison to cochleae cultured with LPS but not ATP (6). Furthermore, intracellular pro-IL-1β expression was elevated in some cochlear *Cx3cr1*^+^ cells in response to LPS stimulation (Figure 3). Mature IL-1β could not be detected in this experiment since it is secreted extracellularly. These findings indicate that macrophage/monocyte-like cells in the cochlea are cells in which the NLRP3 inflammasome exists and can be activated. Thus, these data indicate that some macrophage/monocyte-like cells in the cochlea can be associated with an innate immune response and hearing loss.

## Cochlear Inflammation and Hearing Loss Fluctuation in a Mouse Model of Pendred Syndrome

The stria vascularis is located in the lateral wall of the cochlea and is composed of three layers of cells: marginal cells, intermediate cells, and basal cells. The stria vascularis contains an extensive vascular network that is physiologically compartmentalized from surrounding strial cells by the blood-labyrinth barrier. The blood-labyrinth barrier includes a layer of endothelial cells connected by tight junctions within the walls of strial blood vessels. The stria vascularis produces endolymph and generates the endocochlear potential that is required for inner ear sensory hair cell function. The normally functioning tight junction-based blood-labyrinth barrier is believed to prevent or restrict the diffusion of immune cells, inner ear antigens, and antibodies between the circulation and the strial tissue (26, 27). Macrophages also exist adjacent to or near blood vessels in the stria vascularis, and have been referred to as perivascular macrophage/monocyte-like cells in some publications (28, 29). The integrity of the blood-labyrinth barrier in the stria vascularis is thought to be maintained by vascular endothelial cells, intermediate cells and perivascular macrophages. In fact, depletion of perivascular macrophage by gene targeting produced leaky capillaries and elevated hearing thresholds *in vivo* (30).

**Figure 4:**
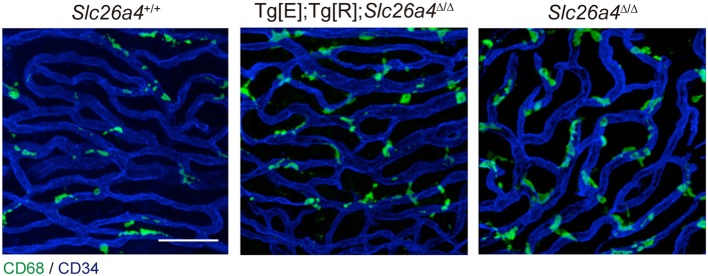
Whole-mount preparation of stria vascularis at 1 month of age stained with anti-CD68 (green) and anti-CD34 antibodies (blue). Anti-CD68 antibodies stain cells of the macrophage–monocyte lineage in control (*Slc26a4*^+/+^; left), *Slc26a4-*insufficient (Tg[E];Tg[R];*Slc26a4*^Δ/Δ^; middle) and *Slc26a4-*null (*Slc26a4*^Δ/Δ^; right) stria vascularis. The area of anti-CD68 staining was higher in *Slc26a4-*insufficient ears compared to control ears, and the area of staining was even higher in *Slc26a4-*null ears. *Slc26a4-*insufficient (Tg[E];Tg[R];*Slc26a4*^Δ/Δ^) mice have the effector transgene (Tg[E]) and the responder transgene (Tg[R]). All *Slc26a4* expression is under the control of doxycycline (33). Blood vessels were stained with anti-CD34 antibodies (blue). Scale bar, 40 μm. Reproduced from Figure 7, Ito et al. (32).

**Figure 5:**
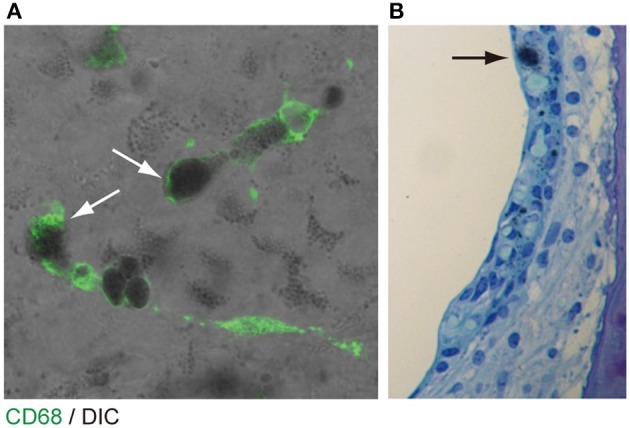
Pigmentation granules and macrophages in the stria vascularis. **(A)** Whole mount preparation of P30 *Slc26a4-*insufficient (Tg[E];Tg[R];*Slc26a4*^Δ/Δ^) stria vascularis stained with anti-CD68 antibody (green). Many pigmentations granules of variable size and shape were observed, some of which were associated with macrophages labeled by CD68 antibody (arrows). Macrophages appear to phagocytose parts of aggregated pigmentation and degenerated intermediate cells. **(B)** P30 *Slc26a4-*insufficient cochlear section stained with toluidine blue. Pigmentation is localized in the intermediate cell layer. *Slc26a4-*insufficient (Tg[E];Tg[R];*Slc26a4*^Δ/Δ^) mice have the effector transgene (Tg[E]) and the responder transgene (Tg[R]). All *Slc26a4* expression is under the control of doxycycline (33). Reproduced from Figure 9, Ito et al. (32).

The origin of the perivascular macrophages in the normal resting stria vascularis is controversial (30, 31). However, significant macrophage invasion or proliferation or both are observed in the *Slc26a4*-null mouse, a model of Pendred syndrome (31). We characterized macrophages in an *Slc26a4*-insufficient mouse with fluctuation of hearing and endocochlear potential (32). The area of staining with anti-CD68 antibodies in the stria vascularis was correlated with the severity of hearing loss (Figure 4). Also, there was a strong correlation of click ABR thresholds with *Cd68* mRNA levels. These data support the hypothesis that macrophage activity is affecting hearing by influencing the function of the stria vascularis. Additionally, we observed that macrophages appeared to phagocytose pigment granules, which are increased in the stria vascularis of *Slc26a4*-insufficient cochleae (Figure 5). The roles of hyperpigmentation and macrophage invasion, activation or proliferation within the stria vascularis of the *Slc26a4*-insufficient cochlea remain undetermined. However, similar observations were reported for macrophages in the iris and ciliary body of the anterior chamber of the eye, presumably to ensure excess pigment granules are retained in the tissue and not released into the anterior chamber of the eye (34).

## Conclusion

Macrophage-like cells are scattered throughout all cochlear tissues, including the auditory nerve, spiral ganglion, basilar membrane, stria vascularis, and spiral ligament. The p.Arg918Gln mutation in *NLRP3* can cause non-syndromic sensorineural hearing loss as well as an atypical presentation of CAPS. In wild-type mouse cochlea, the NLRP3 inflammasome exists and can be activated in the macrophages. These results support the hypothesis that local cochlear activation of the NLRP3 inflammasome can induce cochlear autoinflammation and sensorineural hearing loss in the affected subjects. In an *Slc26a4*-insufficient mouse model of sensorineural hearing loss, macrophage activity is likely affecting hearing by influencing the function of the stria vascularis. Thus, macrophages in the cochlea can affect cochlear and auditory function in more than one disorder and, possibly, in many disorders of hearing.

## Author Contributions

All authors listed have made a substantial, direct and intellectual contribution to the work, and approved it for publication.

### Conflict of Interest

The authors declare that the research was conducted in the absence of any commercial or financial relationships that could be construed as a potential conflict of interest.
